# The Prevalence of Positional Obstructive Sleep Apnoea in a Sample of the Saudi Population

**DOI:** 10.1007/s44197-023-00089-1

**Published:** 2023-01-27

**Authors:** Siraj O. Wali, Ibrahim AlQassas, Sultan Qanash, Hani Mufti, Malak Alamoudi, Maha Alnowaiser, Reem Bakraa, Abdullah Alharbi, Wejdan Ossra, Faris Alhejaili, Ranya Alshumrani, Ghadah A. Batawi

**Affiliations:** 1 Sleep Medicine and Research Center, Sleep Medicine Research Group, Internal Medicine Department, Faculty of Medicine, College of Medicine, King Abdulaziz University Hospital, King Abdulaziz University, PO BOX 21589, Jeddah, 80215 Saudi Arabia; 2 Saudi German Hospital, Jeddah, Saudi Arabia; 3 Internal Medicine Department, National Guard Hospital, King Abdulaziz Medical City, Jeddah, Saudi Arabia; 4 King Abdullah International Medical Research Center, Jeddah, Saudi Arabia; 5 College of Medicine, King Saud Bin Abdulaziz University for Health Sciences, Jeddah, Saudi Arabia; 6 King Faisal Cardiac Center, Ministry of National Guard Health Affairs, Western Region, Jeddah, Saudi Arabia; 7 Faculty of Medicine, King Abdulaziz University, Jeddah, Saudi Arabia; 8 Sleep Medicine Center, National Guard Hospital, King Abdulaziz Medical City, Jeddah, Saudi Arabia

**Keywords:** OSA, POSA, Definition, Prevalence, Predictors

## Abstract

**Purpose:**

Positional obstructive sleep apnoea (POSA) is of important clinical significance, as positional treatment can augment or obviate continuous positive airway pressure. This study aimed to determine the prevalence of POSA and its characteristics using different definitions.

**Methods:**

We retrospectively examined a cohort of patients who underwent polysomnography (PSG) between 2013 and 2019 at two sleep centres. Demographic data and PSG data were collected from 624 patients with an apnoea–hypopnea index (AHI) ≥ 5. POSA was defined using different criteria as follows: (1) AHI of at least twice as high in the supine position as in the lateral position (Cartwright’ s definition). (2) A supine AHI ≥ 10 and a lateral AHI < 10 (Marklun’s definition). (3) AHI of at least twice as high in the supine position than in the lateral position, with the lateral AHI not exceeding 5 (Mador’s definition or Exclusive POSA; e-POSA). (4) AHI ≥ 15/h; a supine AHI ≥ twice that of the nonsupine AHI ≥ 20 min of sleep in the supine and nonsupine positions; and a nonsupine AHI < 15 (Bignold’s definition).

**Results:**

The prevalence of POSA was 54% (Cartwright), 38.6% (Mador), 33.8% (Marklund) and 8.3% (Bignold). Multivariate regression analysis showed a body mass index (BMI) < 35 kg/m^2^ was the only significant predictor of POSA. Mador’s definition had the highest diagnostic yield (sensitivity 63%; specificity 100%; area under the receiver operating characteristic curve 90.2%).

**Conclusion:**

POSA is common, but its prevalence depends on the definition used. Low BMI was identified as a significant predictor.

## Introduction

Sleep-disordered breathing (SDB) is a spectrum of sleep disorders characterized by abnormal breathing during sleep; the most common type of SDB, obstructive sleep apnoea (OSA), affects more than 85% of SDB patients [1]. The prevalence of OSA among American adults is estimated to be 37% (170 million people), whereas in Saudi Arabia, OSA is estimated to affect 8.8% of adults [2, 3]. When left untreated, OSA can increase the risk of hypertension, diabetes and cardiovascular diseases [4]. OSA treatment is also important for the management of hypertension, atrial fibrillation, congestive heart failure and neurological conditions, including epilepsy and stroke [5].

OSA can be subdivided into positional obstructive sleep apnoea (POSA) and nonpositional sleep apnoea (non-POSA) based on an individual’s predominant sleeping position. POSA, also known as supine predominant sleep apnoea, is a condition in which patients exhibit an increased rate of respiratory events specifically during sleep in the supine position [6]. The apnoea–hypopnea index (AHI) measures the average number of apnoea and hypopnea events per hour during sleep and is used to diagnose OSA and classify its severity [7]. In 1984, Cartwright suggested a unique phenotype of OSA that was positional and associated with an AHI that was at least twice as high in the supine position than in the lateral position [8]. This was followed by three other definitions of POSA by Marklund, Mador and Bignold, resulting in a discrepancy in the reported prevalence of POSA in the literature [9]. Overall, POSA seems to be common and is predominantly seen in patients with mild OSA. A recent Swiss study of 1719 subjects in the general population estimated the prevalence of OSA and POSA to be 71% and 53%, respectively [10].

Continuous positive airway pressure (CPAP) is effective in treating OSA by preventing airway collapse, and if used daily for at least six hours, it can decrease sleepiness, improve daily functioning and restore memory to normal levels [11]. Unfortunately, 46–83% of patients may not be compliant with more than four hours of CPAP due to associated side effects, which include nasal symptoms and xerostomia [11]. On the other hand, positional therapy (PT) in those with POSA may augment and sometimes replace conventional CPAP and may even be curative in patients with mild OSA [12]. PT and lifestyle modification (LM) are effective tools for treating OSA; however, they are underutilized in clinical practice [13, 14]. The paucity of randomized controlled trials to support PT combined with the different definitions of POSA and the ongoing search for clinical predictors to guide patient selection might make it challenging for guidelines to strongly recommend PT [12]. There is an increasing body of evidence to support PT as an effective strategy in treating OSA, especially mild to moderate OSA [12, 15]. PT also effectively lowers AHI and reduces CPAP pressures, and it was found to be equivalent to CPAP in patients with POSA [16–18]. Furthermore, patients with POSA and e-POSA had a significantly lower likelihood of treatment adherence (PAP daily use ≥ 4 h) at 6 months and were at higher risk of PAP treatment withdrawal than those without POSA [19]. Heinzer et al. [10] reported in a large population-based study that POSA accounted for 75% of OSA subjects, while e-POSA was present in 36% of OSA subjects, recommending that a large proportion of OSA patients could be treated with PT and again underscoring the importance of establishing the diagnosis of POSA. Furthermore, Oksenberg et al. [15] reported that 35.3% of severe OSA patients had POSA. A total of 75.7% of these patients reported significant improvement with postural therapy by adopting the lateral posture. Moreover, nearly one-fifth of patients (18.2%) gained more benefit from postural therapy than from standard CPAP therapy. These data support the efficacy of postural therapy even in severe cases of POSA. This again emphasizes the importance of determining the phenotype status of OSA, particularly in those who cannot tolerate CPAP therapy.

Hence, identifying POSA as a phenotype may play an important role in the management of patients with OSA. Accordingly, the purpose of this study was to evaluate the prevalence and clinical predictors of POSA in a sample of the Saudi population using the common available definitions.

## Materials and Methods

### Study Design and Setting

This retrospective cohort study was conducted at the Sleep Medicine and Research Center (SMRC) at King Abdulaziz University Hospital (KAUH) and King Abdulaziz Medical City (KAMC), National Guard Health Affairs in Jeddah, Saudi Arabia. The study was approved by the Institutional Review Boards (IRBs) of KAUH and KAMC.

### Study Population

All adult patients aged > 18 years referred to SMRC or KAMC who underwent complete polysomnography (PSG) between 2013 and 2019 were included in the study. In all studies, patients must have slept on supine and nonsupine positions. Patient with central sleep apnea or AHI of less than 5 were excluded. In addition, those diagnosed using a split night protocol were also excluded. The records of 379 patients from SMRC, KAUH and 245 patients from KAMC fulfilled the above criteria.

### Data Collection Instruments

Patients’ medical records were reviewed for demographic data [age, sex, body mass index (BMI)]; Epworth Sleepiness Scale (ESS) score [20], which is a self-administered questionnaire routinely used to assess daytime sleepiness; and polysomnographic data. The scoring was standardized by following the American Academy of Sleep medicine (AASM) guidelines. In both centres, certified sleep technologists scored PSG records manually and certified sleep physicians reviewed them in accordance with AASM scoring rules [21]. All data were entered and configured using Microsoft Excel (2016).

In our study, the diagnosis of sleep apnoea was based on full polysomnography when the AHI was ≥ 5 events per hour of sleep [21]. POSA was defined in four different ways:

1. An AHI that was at least twice as high in the supine position than in the lateral position [8].
2. A supine AHI ≥ 10, together with a lateral AHI < 10 [22].
3. An AHI that was at least twice as high in the supine position than in the lateral position, but with a lateral AHI not exceeding 5 [6]; this is also called exclusive POSA (e-POSA).
4. An overall AHI ≥ 15; a supine AHI ≥ twice that of the nonsupine AHI ≥ 20 min of sleep in the supine and nonsupine positions; and a nonsupine AHI < 15 [23].

Furthermore, the following parameters were obtained from the polysomnographic data:

- AHI: (number of apnoea events + number of hypopnoea events)/total sleep time (h)
- Supine AHI: (number of apnoea events + number of hypopnoea events) while in the supine position/total sleep time (h) in the supine position
- Nonsupine AHI: (Number of apnoea events + number of hypopnea events) while in the nonsupine position/total sleep time (h) in the nonsupine position
- AHI in REM sleep: (number of apnoea events + number of hypopnoea events)/total sleep time (h) in REM sleep
- AHI in non-REM sleep: (number of apnoea events + number of hypopnoea events)/total sleep time (h) in non-REM sleep
- Time in bed (TIB): total time spent in bed from the lights off and lights on markers
- Total sleep time (TST): period of sleep time between the lights off and lights on markers, excluding all wake stages.
- Sleep efficiency (%): TST/TIB.
- Mean O_2_ saturation: average value of the complete SpO_2_ curve.
- Time spent with O_2_ saturation less than 90%: percentage of sleep time with oxygen saturation < 90%.

### Statistical Analysis

Several characteristics of patients who developed POSA were compared to patients who did not develop POSA. For continuous variables, we started by assessing whether they fit a normal distribution using the visual approach (density plot and quantile‒quantile plot) and the Shapiro‒Wilk method. The mean and standard deviation were used for continuous variables with normal distribution. The median and interquartile range were used for continuous variables that were not normally distributed. To compare the continuous variables, we used either the Welch two-sample *t* test or the Wilcoxon rank sum test. For the categorical variables, frequencies and percentages were used. To evaluate the associations between the categorical variables, we applied the Chi-square or Fisher’s exact test. The Kruskal–Wallis test was applied for ordinal variables.

We initially performed an ANOVA to ascertain the overall differences between the groups. Then, variables with *p* values indicating significance in the ANOVA testing were further examined with the pairwise t test accounting for multiple testing with a Bonferroni correction of the *p* value. Because of the small number of comparisons (less than 5), we elected to use the Bonferroni correction; it is more conservative, which should reduce the false positive rate. Univariate and bivariate analyses were used to identify risk factors that influence the development of POSA based on each definition. Binary logistic regression was then used to evaluate the influence of several independent risk factors on the development of POSA based on each definition. This was reported using odds ratios (ORs) with 95% confidence intervals (CIs).

Sensitivity analysis was performed to compare the general performance of each definition of POSA compared to the standard definition (Definition 1) as the gold standard. The sensitivity, specificity, positive predictive value (PPV), negative predictive value (NPV) and overall accuracy were compared. To assess the predictive accuracy of each definition, the area under the receiver operating characteristic curve (AUROC) with standard error and 95% CIs was calculated. Because there is no gold standard definition that is accepted for POSA, we compared each definition with all the other definitions. All statistical tests were two-tailed, and *p* values < 0.05 were considered to indicate significance. All statistical analyses were performed using R software, version 4.0.2 [24].

## Results

### Patient Characteristics

The study cohort included 624 patients. The mean age was 50.2 years (SD 13.7), the mean BMI was 36.6 kg/m^2^ (SD 9.6), and 52.08% of the patients were male. The mean ESS score of the available data was 11.1 (SD = 5.6). Medical comorbidities were also obtained from patients’ records (Table 1).

**Table 1:** General and sleep-related characteristics of the study population

Variable	Full dataset (*n* = 624)
Age in years, mean (SD)	50.2 (13.7)
Male sex, *n* (%)	325 (52.08)
BMI in kg/m^2^, mean (SD)	36.6 (9.6)
DM, *n* (%)	205 (32.85)
COPD, *n* (%)	230 (36.8)
Asthma, *n* (%)	244 (39.1)
HTN, *n* (%)	308 (49.4)
IHD, *n* (%)	259 (41.5)
Epworth Sleepiness Scale score, mean (SD)	11.1 (5.6)
TST (min), mean (SD)	268.1 (66.2)
Sleep efficiency (%), mean (SD)	69.7 (16.5)
Non-REM minutes, mean (SD)	227 (54.6)
REM minutes, mean (SD)	39.1 (23.3)
Time spent in supine position (min), mean (SD)	10.7 (28.8)
AHI, mean (SD)	22.2 (17)
AHI in REM sleep, mean (SD)	36.5 (21.5)
AHI in non-REM sleep, mean (SD)	19.4 (17.7)
AHI in the supine position, mean (SD)	25.4 (20.8)
AHI in the nonsupine position, mean (SD)	14 (16.3)
Mean O_2_ saturation, mean (SD)	94.7 (2.3)
Minimum O_2_ saturation, mean (SD)	50.2 (39.8)
Time spent < 90% O_2_ saturation (min), mean (SD)	7 (14.3)

*BMI* body mass index, *ESS* Epworth Sleepiness Scale, *DM* diabetes mellitus, *COPD* chronic obstructive pulmonary disease, *HTN* hypertension, *IHD* ischaemic heart disease, *TST* total sleep time, *TIB* time in bed, *REM* rapid eye movement, *AHI* apnoea–hypopnea index

### Sleep-Related Characteristics of the Full Population

The polysomnographic data of all 624 patients were reviewed. The mean AHI was 22.2 (SD 17). The mean TIB and TST were 387.17 min (SD 45.9) and 268.1 min (SD 66.2), respectively (Table 1).

### Characteristics of Patients Based on POSA Definition 1

Using the standard definition (Definition 1), the patients were divided into two groups: 46% of patients met the definition of non-POSA (non-POSA-Def 1), and 54% of patients met the definition of POSA (POSA-Def 1) Table 2.

**Table 2:** General characteristics of patients classified according to POSA Definition 1

Patient characteristics	All patients (*n* = 624)	Non-POSA* (*n* = 287, 46%)	POSA** (*n* = 337, 54%)	*p* value
Age (years), mean (SD)	50.2 (13.7)	51.3 (13.9)	49.3 (13.5)	0.28
Male sex, *n* (%)	325 (52.08)	137 (47.7)	188 (55.8)	0.045*
BMI (kg/m^2^), mean (SD)	36.6 (9.6)	38.1 (8.5)	34.2 (7.7)	< 0.001*
DM, *n* (%)	205 (32.85)	83 (36.8)	123 (43.6)	0.12
COPD, *n* (%)	230 (36.8)	105 (47.3)	125 (44.2)	0.484
Asthma, *n* (%)	244 (39.1)	108 (47.6)	136 (47.2)	0.936
HTN, *n* (%)	308 (49.4)	87 (36.2)	123 (44.2)	0.0646
IHD, *n* (%)	259 (41.5)	124 (52.3)	135 (48)	0.332
Epworth Sleepiness Scale score, mean (SD)	11.1 (5.6)	11.7 (5.8)	10.8 (6.1)	0.211
AHI, mean (SD)	22.2 (17)	25.5 (19.2)	19.5 (14.4)	< 0.001*
AHI in REM sleep, mean (SD)	36.5 (21.5)	40.7 (22)	33 (20.5)	< 0.001*
AHI in the supine position, mean (SD)	25.4 (20.8)	22.5 (22.8)	27.7 (18.9)	< 0.001*
Mean O_2_ saturation, mean (SD)	94.7 (2.3)	94.4 (2.4)	95 (2.2)	0.061
Time spent < 90% O_2_ saturation, mean (SD)	7 (14.3)	8.7 (16)	5.6 (12.5)	0.009*

*p value of <0.05 is considered to be significant

Males were more likely than females to suffer from POSA-Def 1 (55.8%, *p* value < 0.001). Patients with POSA-Def 1 had lower BMIs than patients with non-POSA-Def 1 (32.9 kg/m^2^ and 34.2 kg/m^2^ vs*.* 38.1 kg/m^2^, *p* value < 0.001). The time spent with an oxygen saturation of less than 90% during sleep was significantly shorter in patients with POSA-Def 1 than in patients with non-POSA-Def 1 (5.6 *vs.* 8.7 min, *p* value = 0.009). There was no significant difference between POSA-Def 1 patients and non-POSA-Def 1 patients in terms of comorbidities (Table 2). Based on the univariate logistic regression assessment of significant predictors of POSA, four variables were predictors: BMI ≤ 35 kg/m^2^ with an OR 1.76 (95% CI 1.27–2.44, *p* value < 0.001), male sex with an OR 1.38 (95% CI 1.01–0.1.89, *p* value = 0.0451), AHI > 10 with an OR 0.63 (95% CI 0.44–0.9, *p* value = 0.0123), and an AHI in REM > 20 with an OR 0.64 (95% CI 0.44–0.92, *p* value = 0.1717) (see Table 3 for all definitions in the univariate logistic regression analysis).

**Table 3:** Univariate analysis of significant variables using logistic regression

Variable	Units	Def 1			Def 2			Def 3		
		OR	CI 95%	*p* value	OR	CI 95%	*p* value	OR	CI 95%	*p* value
Age in years	≤ 50	Ref			Ref			Ref		
	> 50	0.87	[0.63;1.21]	0. 4134	1.23	[0.88;1.71]	0.2209	0.75	[0.53;1.05]	0.0922
Gender	Female	Ref			Ref			Ref		
	Male	1.38	[1.01;1.89]	0.04505	1.57	[1.13;2.17]	0.0068	1	[0.72;1.40]	0.9859
BMI in kg/m^2^	> 35	Ref			Ref			Ref		
	≤ 35	1.76	[1.27;2.44]	< 0.001	0.63	[0.45;0.88]	0.0061	1.52	[1.08;2.15]	0.0165
DM	No	Ref			Ref			Ref		
	Yes	1.33	[0.93;1.91]	0.1202	0.73	[0.50;1.06]	0.0949	1.8	[1.24;2.60]	0.0019
COPD	No	Ref			Ref			Ref		
	Yes	0.88	[0.62;1.25]	0.4837	1.12	[0.78;1.61]	0.5426	1	[0.69;1.44]	0.9971
Asthma	No	Ref			Ref			Ref		
	Yes	0.99	[0.70;1.40]	0.9362	0.95	[0.66;1.36]	0.785	0.99	[0.69;1.43]	0.974
HTN	Yes	Ref			Ref			Ref		
	No	0.72	[0.50;1.02]	0.06495	1.03	[0.72;1.48]	0.8733	0.60	[0.42;0.87]	0.0069
IHD	Yes	Ref			Ref			Ref		
	No	1.19	[0.84;1.68]	0.3321	0.88	[0.61;1.25]	0.4687	1.1	[0.70;1.44]	0.999
AHI	< 10	Ref			Ref			Ref		
	> 10	0.63	[0.44;0.90]	0.0123	92.56	[22.67;377.89]	< 0.001	3.41	[2.36;4.93]	< 0.001
AHI in REM	≤ 20	Ref			Ref			Ref		
	> 20	0.64	[0.44;0.92]	0.01717	5.13	[3.19;8.26]	< 0.001	3.01	[2.12;4.27]	< 0.001
Mean SaO_2_ in %	≤ 95	Ref			Ref			Ref		
	> 95	1.42	[0.93;2.18]	0.1061	0.55	[0.35;0.86]	0.0083	1.08	[0.69;1.68]	0.7482
Time SaO_2_ Less 90%	≤ 2	Ref			Ref			Ref		
	> 2	0.65	[0.46;0.92]	0.01386	1.97	[1.39;2.79]	< 0.001	0.65	[0.45;0.94]	0.0223

*BMI* body mass index, *DM* diabetes mellites, *COPD* chronic obstructive pulmonary disease, *HTN* hypertension, *IHD* ischaemic heart disease, *AHI* apnoea–hypopnea index, *SaO*_*2*_ oxygen saturation, *REM* rapid eye movement

### Characteristics of Patients Based on POSA Definition 2

Using Definition 2, the patients were divided into two groups: 61.4% met the definition of non-POSA (non-POSA-Def 2), and 38.6% met the definition of POSA (POSA-Def 2) Table 4.

**Table 4:** General characteristics of patients classified according to POSA Definition 2

Patient characteristics	All patients (*n* = 624)	Non-POSA (*n* = 383, 61.4%)	POSA (*n* = 241, 38.6%)	*p* value
Age (years), mean (SD)	50.2 (13.7)	49.8 (14)	51 (13.1)	0.934
Male sex, *n* (%)	325 (52.08)	183 (47.8)	142 (58.9)	0.007*
BMI (kg/m^2^), mean (SD)	36.6 (9.6)	34.9 (8.3)	37.7 (7.95)	< 0.001*
DM, *n* (%)	205 (32.85)	138 (43.4)	67 (35.8)	0.945
COPD, *n* (%)	230 (36.8)	142 (44.5)	88 (47.3)	0.543
Asthma, *n* (%)	244 (39.1)	155 (47.8)	89 (46.6)	0.785
HTN, *n* (%)	308 (49.4)	131 (40.8)	79 (40.1)	0.873
IHD, *n* (%)	259 (41.5)	157 (48.8)	102 (52)	0.496
Epworth Sleepiness Scale score, mean (SD)	11.1 (5.6)	10.5 (5.7)	11.9 (5.3)	0.026*
AHI, mean (SD)	22.2 (17)	15.9 (13.2)	32.6 (17.5)	< 0.001*
AHI in REM sleep, mean (SD)	36.5 (21.5)	29.5 (19.6)	48.1 (19.5)	< 0.001*
AHI in the supine position, mean (SD)	25.4 (20.8)	16.5 (15)	40.3 (20.6)	< 0.001*
Mean O_2_ saturation, mean (SD)	94.7 (2.3)	94.9 (2.29)	94.5 (2.22)	0.168
Time spent < 90% O_2_ saturation, mean (SD)	7 (14.3)	5.8 (13.3)	8.9 (15.6)	0.012*

*p value of <0.05 is considered to be significant

Males were still more likely to suffer from POSA-Def 2 (58.9%, *p* value < 0.001). Patients with POSA-Def 2 had a lower BMI than patients with non-POSA-Def 2 (32.9 kg/m^2^ and 34.9 kg/m^2^ *vs.* 37.7 kg/m^2^, *p* value < 0.001). There was no significant difference between POSA-Def two patients and non-POSA-Def 2 patients in terms of comorbidities. The time spent with a oxygen saturation of less than 90% during sleep was significantly longer in patients with POSA-Def 2 than in patients with non-POSA-Def 2 (8.7 vs. 5.6 min, *p* value = 0.012) (Table 4). Based on the univariate logistic regression assessment of significant predictors of POSA based on definition 2, six variables were associated with POSA: male sex with an OR 1.57 (95% CI 1.13–2.17, *p* value = 0.0068), BMI ≤ 35 kg/m^2^ with an OR 0.63 (95% CI 0.45–0.88, *p* value = 0.0061), AHI > 10 with an OR 92.6 (95% CI 22.7–377.9, *p* value < 0.001), AHI in REM > 20 with an OR 5.13 (95% CI 3.19–8.26, *p* value < 0.001), mean oxygen saturation > 95% with an OR 0.55 (95% CI = 0.35–0.86, *p* value = 0.008), and sleep time with an SaO_2_ less than 90% with an OR 1.97 (95% CI 1.39–2.79, *p* value < 0.001) (see Table 3 for all definitions in the univariate logistic regression analysis).

### Characteristics of Patients Based on POSA Definition 3

Using Definition 3, the patients were divided into two groups: 66.2% met the definition of non-POSA (non-POSA-Def 3), and 33.8% met the definition of POSA (POSA-Def 3) Table 5.

**Table 5:** General characteristics of patients classified according to POSA Definition 3

Patient characteristics	All patients (*n* = 624)	Non-POSA (*n* = 413, 66.2%)	POSA (*n* = 211, 33.8%)	*p* value
Age (years), mean (SD)	50.2 (13.7)	50.9 (13.5)	49 (13.9)	0.029*
Male sex, *n* (%)	325 (52.08)	215 (52.1)	110 (52.1)	0.986
BMI (kg/m^2^), mean (SD)	36.6 (9.6)	37 (8.3)	34.1 (7.9)	< 0.001*
DM, *n* (%)	205 (32.85)	115 (35.5)	90 (49.7)	0.002*
COPD, *n* (%)	230 (36.8)	148 (45.5)	82 (45.6)	0.997
Asthma, *n* (%)	244 (39.1)	157 (47.4)	87 (47.3)	0.974
HTN, *n* (%)	308 (49.4)	123 (36.3)	87 (48.6)	0.007*
IHD, *n* (%)	259 (41.5)	170 (50)	89 (50)	1
Epworth Sleepiness Scale score, mean (SD)	11.1 (5.6)	11.3 (5.38)	10.6 (5.93)	0.698
AHI score, mean (SD)	22.2 (17)	27.6 (21.4)	18.9 (19.9)	< 0.001*
AHI score in REM sleep, mean (SD)	36.5 (21.5)	39.7 (21.8)	30.3 (19.6)	< 0.001*
AHI in the supine position, mean (SD)	25.4 (20.8)	27.9 (23)	20.6 (14.6)	< 0.001*
Mean O_2_ saturation, mean (SD)	94.7 (2.3)	94.7 (2.2)	94.8 (2.4)	1
Time spent < 90% O_2_ saturation, mean (SD)	7 (14.3)	7.36 (14.6)	6.2 (13.6)	0.829

*p value of <0.05 is considered to be significant

Patients with POSA-Def 3 were younger than patients with non-POSA-Def 3 (49 years vs. 50.9 years, *p* value = 0.029). There was no male predominance as in the first two definitions. Patients with POSA-Def 3 had lower BMIs than patients with non-POSA-Def 3 (34.1 kg/m^2^ vs. 37 kg/m^2^, *p* value < 0.001). Based on Definition 3, diabetes mellitus (DM) and hypertension became statistically significant predictors (*p* value = 0.002 and 0.007, respectively), with a lower distribution in the non-POSA-Def 3 group (35.5% compared to 49.7% for DM, and 36.3% compared to 48.6% for hypertension in the POSA-Def 3 group). The time spent with an oxygen saturation of less than 90% during sleep was not significantly different in patients with POSA-Def 3 compared to patients with non-POSA-Def 3 (7.4 vs. 6.2 min, *p* value = 0.829). Based on the univariate logistic regression assessment of significant predictors of POSA, six variables were associated with POSA: BMI less than or equal to 35 kg/m^2^ with an OR = 1.52 (95% CI 1.08–2.15, *p* value = 0.017), history of DM with an OR 1.8 (95% CI 1.24–2.6, *p* value = 0.0019), history of hypertension with an OR 0.6 (95% CI 0.42–0.87, *p* value = 0.007), AHI > 10 with an OR 3.42 (95% CI 2.36–4.93, *p* value < 0.001), AHI in REM > 20 with an OR 3.01 (95% CI 2.12–4.27, *p* value < 0.001), and a time spent with an oxygen saturation less than 90% during sleep of more than 2 min with an OR 0.65 (95% CI 0.45–0.94, *p* value = 0.223) (see Table 3 for all definitions in the univariate logistic regression analysis).

### Characteristics of Patients Based on POSA Definition 4

Using Definition 4, the patients were further divided into two groups: 91.7% of patients met the definition of non-POSA (non-POSA-Def 4), and 8.3% of patients met the definition of POSA (POSA-Def 4) Table 6.

**Table 6:** General characteristics of patients classified according to POSA Definition 4

Patient characteristics	All patients (*n* = 624)	Non-POSA (*n* = 572, 91.7%)	POSA (*n* = 52, 8.3%)	*p* value
Age (years), mean (SD)	50.2 (13.7)	50.1 (13.8)	51.8 (12.9)	1
Male sex, *n* (%)	325 (52.08)	292 (51)	33 (63.5)	0.086
BMI (kg/m^2^), mean (SD)	36.6 (9.6)	36.2 (8.4)	34 (6.6)	0.214
DM, *n* (%)	205 (32.85)	187 (40.5)	18 (41.9)	0.86
COPD, *n* (%)	230 (36.8)	213 (46.1)	17 (39.5)	0.408
Asthma, *n* (%)	244 (39.1)	230 (48.8)	14 (31.8)	0.031*
HTN, *n* (%)	308 (49.4)	189 (39.6)	21 (51.2)	0.147
IHD, *n* (%)	259 (41.5)	241 (50.7)	18 (41.9)	0.265
Epworth Sleepiness Scale score, mean (SD)	11.1 (5.6)	11.1 (5.61)	10.3 (5.32)	1
AHI, mean (SD)	22.2 (17)	21.6 (17.4)	25 (10.8)	0.424
AHI in REM sleep, mean (SD)	36.5 (21.5)	36.3 (21.6)	38.3 (20.3)	1
AHI in the supine position, mean (SD)	25.4 (20.8)	24.2 (20.8)	38 (15.5)	< 0.001*
Mean O_2_ saturation, mean (SD)	94.7 (2.3)	94.7 (2.3)	95.4 (1.88)	0.149
Time spent < 90% O_2_ saturation, mean (SD)	7 (14.3)	7.18 (14.6)	4.57 (9.6)	0.488

*p value of <0.05 is considered to be significant

Interestingly, in the pairwise comparison, there was no significant difference between POSA-Def 4 patients and non-POSA-Def 4 patients in terms of demographic parameters, oxygenation parameters and comorbidities except for asthma (48.8% in non-POSA *vs.* 31.8% in POSA patients with a *p* value = 0.031) (Table 6). Because of the significant imbalance between positive and negative cases based on Definition 4, a simple univariate logistic regression was noninformative and was not used, as most of the variables had very wide confidence intervals mainly due to the small representation of positive cases in the dataset, which made finding a stable statistical solution impossible for the algorithm.

### Differences Between the Four Sets of Criteria

There was no significant difference between the four sets of criteria with regard to age, sex, or comorbidities except for DM, which was significant in Definition 3, and ESS score, which was significant in Definition 2.

### Sensitivity Analysis of the Four Definitions of POSA

**Fig. 1:**
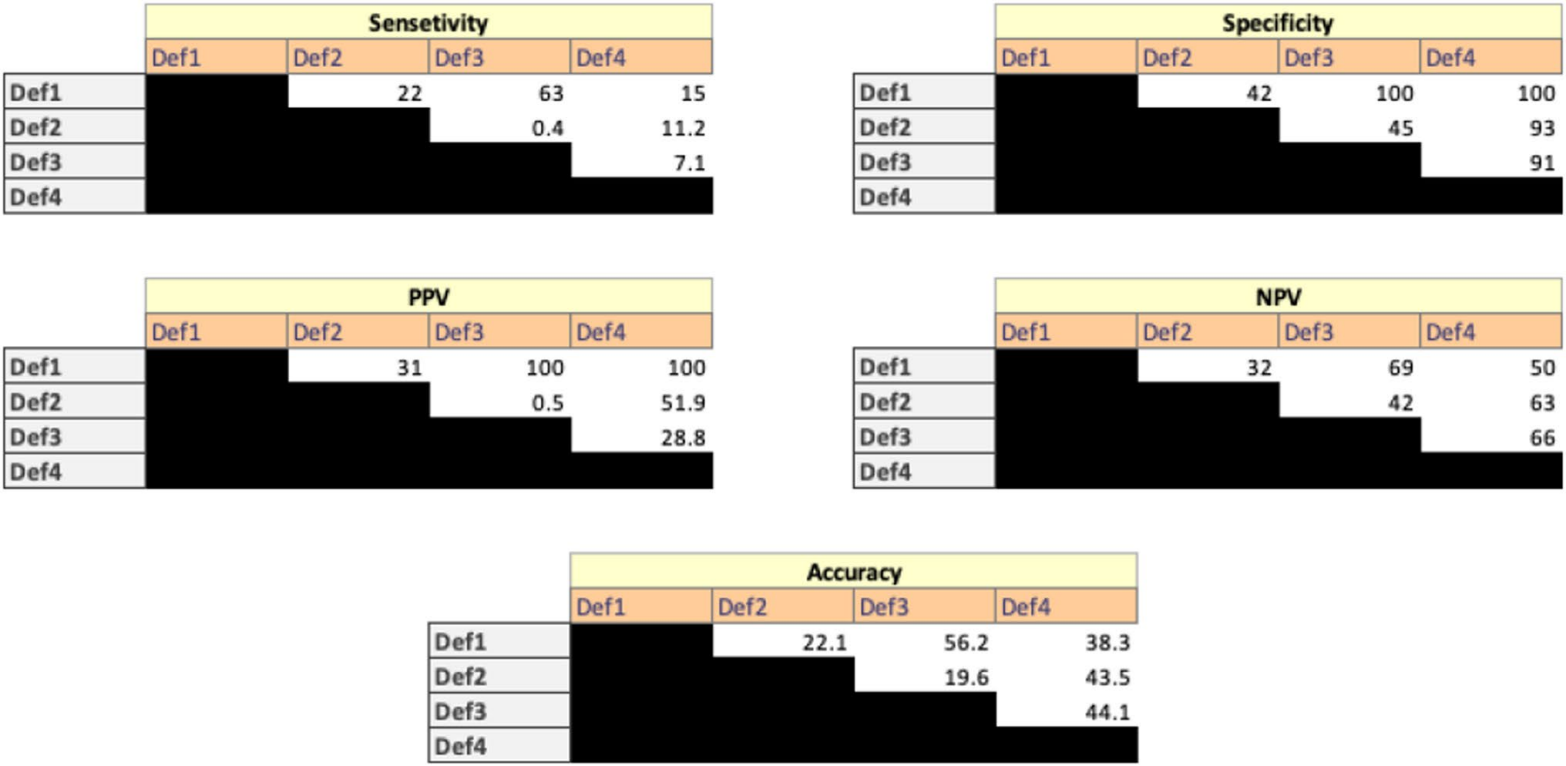
Analysis matrix of all definitions

**Fig. 2:**
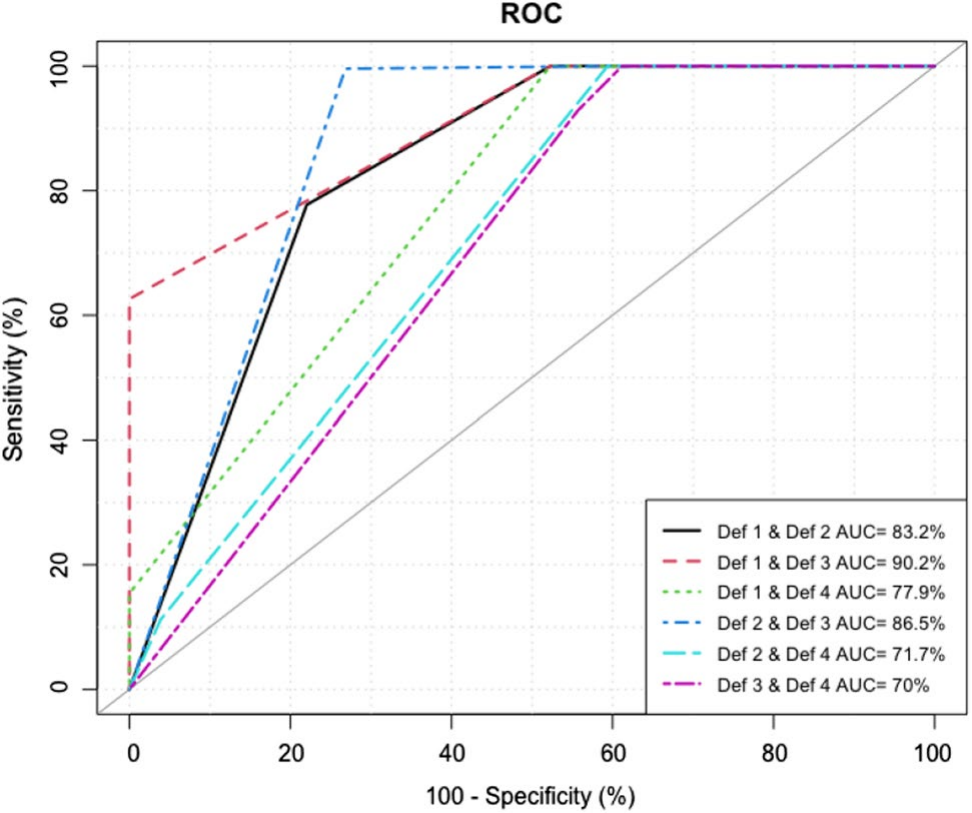
ROC-AUC for all possible definition combinations

Since there is no standard definition for POSA and to determine the performance of each definition, a sensitivity analysis was conducted. Definition 1 was assumed to be the basic standard definition against which the other three definitions were compared. The following parameters were compared: sensitivity, specificity, PPV, NPV and accuracy. Figure 1 depicts the matrix of these comparisons. When assessing different combinations of definitions, starting with one definition as a screening tool, followed by another definition as a confirmatory tool, the combination of Definition 1 as a screening tool and Definition 3 as a confirmatory tool generated the best overall results (sensitivity 63%, specificity 100%, PPV 100%, NPV 69% and overall accuracy 56.2%). Other combinations were significantly worse. Since Definition 1 is part of Definition 3, and based on the sensitivity analysis, Definition 3 has the highest diagnostic yield for patients with POSA. Using the AUROC analysis, Fig. 2 redemonstrates that the combination of Definitions 1 and 3 resulted in the best diagnostic value of patients with POSA (AUC 90.2%).

### Multivariate Logistic Regression

Based on the multivariate logistic regression, all definitions agreed that a BMI less than or equal to 35 kg/m^2^ was the only predictive variable for POSA. All other variables in Definitions 1 and 3 became nonsignificant. For Definition 2, male sex remained a significant predictor, and a mean oxygen saturation > 95% remained a significant negative predictor, but AHI and AHI in REM became nonsignificant. All multivariate models had a good predictive value (AUROC between 77.7 and 88.8%) (see Table 7 for all definitions in the multivariate logistic regression analysis).

**Table 7:** Multivariate analysis using logistic regression

Variable	Units	Def 1			Def 2			Def 3		
		OR	CI 95%	*p* value	OR	CI 95%	*p* value	OR	CI 95%	*p* value
Age in years	≤ 50	Ref			Ref			Ref		
	> 50	0.74	[0.31;1.80]	0.5076	0.98	[0.34;2.83]	0.9679	0.74	[0.31;1.80]	0.5076
Gender	Female	Ref			Ref			Ref		
	Male	1.21	[0.50;2.90]	0.6696	3.17	[1.05;9.53]	0.04	1.21	[0.50;2.90]	0.6696
BMI in kg/m^2^	> 35	Ref			Ref			Ref		
	≤ 35	3.89	[1.60;9.45]	0.0027	0.21	[0.07;0.70]	0.0102	3.89	[1.60;9.45]	0.0027
DM	No	Ref			Ref			Ref		
	Yes	2.53	[0.90;7.11]	0.0784	0.71	[0.21;2.39]	0.5769	2.53	[0.90;7.11]	0.0784
COPD	No	Ref			Ref			Ref		
	Yes	1.16	[0.08;17.23]	0.9160	4,395,779.65	[0.00;Inf]	0.9942	1.16	[0.08;17.23]	0.9160
Asthma	No	Ref			Ref			Ref		
	Yes	0.45	[0.16;1.22]	0.1154	1.64	[0.47;5.71]	0.4376	0.45	[0.16;1.22]	0.1154
HTN	Yes	Ref			Ref			Ref		
	No	0.50	[0.18;1.39]	0.1817	1.98	[0.61;6.42]	0.2540	0.50	[0.18;1.39]	0.1817
IHD	Yes	Ref			Ref			Ref		
	No	3.82	[0.80;18.31]	0.0932	0.31	[0.05;1.91]	0.2078	3.82	[0.80;18.31]	0.0932
AHI	< 10	Ref			Ref			Ref		
	> 10	1.08	[0.41;2.79]	0.8812	0.00	[0.00;Inf]	0.9915	1.08	[0.41;2.79]	0.8812
AHI in REM	≤ 20	Ref			Ref			Ref		
	> 20	0.46	[0.17;1.24]	0.1245	3.63	[1.00;13.27]	0.0508	0.46	[0.17;1.24]	0.1245
Mean SaO_2_	≤ 95	Ref			Ref			Ref		
	> 95	1.31	[0.44;3.86]	0.6283	0.20	[0.05;0.84]	0.0276	1.31	[0.44;3.86]	0.6283
Time SaO_2_ less 90%	≤ 2	Ref			Ref			Ref		
	> 2	0.38	[0.13;1.17]	0.0912	0.77	[0.19;3.15]	0.7158	0.38	[0.13;1.17]	0.0912
ROC-AUC		77.7	[69.5;85.8]		88.8	[83.3;94.4]		80.6	[72.2;88.9]	

## Discussion

Different studies have shown that more than 50% of patients with OSA are likely to have POSA. It was also found that in approximately 80% of OSA patients, the AHI was higher in the supine position than in the nonsupine position. Unfortunately, despite the high prevalence of POSA, clinicians focus mainly on CPAP, and PT is usually ignored. Therefore, in this study, we tried to determine the prevalence of POSA using the four commonly applied definitions and address its positive predictors. The prevalence of POSA in our study was 54% (Definition 1; Cartwright), 38.6% (Definition 2; Marklund), 33.8% (Definition 3; Mador), and 8.3% (Definition 4; Bignold). Based on the sensitivity analysis, the Mador definition had the highest diagnostic yield for POSA, with a sensitivity and specificity of 63% and 100%, respectively. Furthermore, with multivariate regression analysis, a BMI < 35 kg/m^2^ was the only significant predictor of PSA across all applied definitions.

The prevalence of POSA in the literature, as shown in our study, depends primarily on the chosen definition. In our study, we found that with the Cartwright definition, the prevalence of POSA was 54%, which was similar to that reported in several studies. Studies from the United Arab Emirates (UAE), Australia, France, Switzerland and Denmark reported the following prevalence rates of POSA: 53%, 61%, 53.5%, 53% and 62.3%, respectively [10, 19, 25–27]. However, using the Mador definition, we reported a prevalence of 33.8%, which was again close to that reported in the literature. Studies from France, Switzerland and Denmark reported prevalence rates of 20.1%, 26%, and 29.1%, respectively [10, 19, 27].

We went further and tried to identify the predictors of POSA. Our study revealed that male sex, younger age, a lower BMI, time spent with an oxygen saturation less than 90% during sleep, DM, hypertension and a history of asthma were significant factors associated with POSA. However, when a multivariate regression analysis was used, a low BMI of less than 35 kg/m^2^ and male sex remained positive predictors for POSA. It is not clear why low BMI is a predictor of POSA. It is expected that high BMI rather than low BMI will be associated with POSA. High BMI may make the patient symptomatic regardless of the position, and hence it may be difficult to differentiate between symptoms of the patient, regardless of the position during sleep. It is possible that low BMI may be associated with mild OSA, which is only obvious during POSA. Indeed, this link between low BMI, mild OSA and POSA was already reported in the literature and again shown in the current study according to Definition 1 of POSA. Nevertheless, more studies are required to clarify this association. Indeed, our study has shown that an AHI > 10 and an AHI during REM > 20 were associated with a low OR (0.63 and 0.64, respectively), which indicates that the milder the disease, the more likely POSA will develop (Definition 1, Cartwright). The same findings were seen with Definition 2 (Marklund), although the parameters measuring the severity of sleep apnoea showed conflicting results. Moreover, with Definition 3 (Mador), low BMI, DM and severe OSA according to a AHI > 10 and REM AHI > 20 were identified predictors for POSA, while hypertension was identified as a negative predictor for POSA. Similarly, when multivariate regression analysis was applied taking into consideration all other variables, only a low BMI of less than 35 kg/m^2^ remained a predictive variable across all definitions. Compared with other definitions, Marklund (Definition 2) also revealed that male sex and better oxygenation according to a mean oxygen saturation of > 95% remained significant predictors for POSA. Nevertheless, our findings seem to be in agreement with the findings in the literature. Studies have shown that male sex, younger age, lower AHI, lower BMI and time in the supine position are associated with POSA [10, 19, 25]. Moreover, the Mallampati score and heavy alcohol consumption were found to be associated factors in previous studies [10, 25]. Zinchuk et al. [28] also reported that patients with POSA tend to be younger, have a lower BMI, and have lower AHIs than their nonpositional counterparts. Uzer et al. [29] also emphasized that POSA patients have a lower BMI than REM-related OSA patients. In the UAE study, age, BMI, diastolic blood pressure, Mallampati score, and Berlin score were found to be the best predictive factors for POSA, with an AUC of 0.71 (95% CI [0.63, 0.78]) [26]. Oksenberg et al. [15] found that POSA patients were less obese and had less severe OSA (*p* < 0.001) than non-POSA patients among the severe OSA patients they studied. Hence, our study agrees with the literature in that patients with POSA are less obese and have milder disease. Furthermore, our study failed to show a link between POSA and comorbidities regardless of which definition was used. This finding supports the notion that POSA is more likely to be present in patients with relatively less severe OSA. In pure POSA, non-CPAP PT may obviate the need for the use of CPAP. Moreover, in severe OSA with associated elements of POSA, using PT tends to help reduce pressure steering in CPAP.

However, do patients with POSA convert to non-POSA upon follow-up? Oksenberg et al. [30] reported that approximately two-thirds of POSA patients remained in the supine position predominantly upon follow-up for a mean of 6.6 years; however, the remaining patients converted to non-POSA. This information highlights the importance of close follow-up of these patients and that most POSA patients would benefit from postural therapy if they remained compliant with therapy.

This study to our knowledge is the first investigation of the prevalence of POSA using four different commonly applied definitions. The results align with the observed trend that a lower BMI is a positive predictor of POSA. The limitations of our study include its cross-sectional, the retrospective nature, and PT interventions were not studied. Also, it is not multi-centre study and based on hospital based data. Further randomized controlled trials are needed to investigate the positive effects of PT on OSA and to confirm the patient characteristics that are predictive of POSA.

## Conclusion

POSA is common, and its prevalence depends on the definition used. It seems to be associated with male sex, milder disease and a relatively low BMI. It seems that Mador’s definition of e-POSA yields the highest sensitivity, specificity and a stable AUROC. Regardless of the definition used, a lower BMI is a strong predictor of POSA. This finding emphasizes the importance of non-CPAP PT, which is currently relatively underutilized in clinical practice. Nevertheless, POSA remains a common condition, with variable prevalence depending on the definition used.

## Data Availability

Data is available upon request.

## Abbreviations

AHI: Apnoea–hypopnea index
AUROC: Area under the receiver operating characteristic curve
BMI: Body mass index
CIs: Confidence intervals
CPAP: Continuous positive airway pressure
DM: Diabetes mellitus
e-POSA: Exclusive POSA
ESS: Epworth Sleepiness Scale
IRBs: Institutional review boards
KAMC: King Abdulaziz Medical City
KAUH: King Abdulaziz University Hospital
LM: Lifestyle modification
non-POSA: Nonpositional sleep apnoea
NPV: Negative predictive value
ORs: Odds ratios
OSA: Obstructive sleep apnoea
POSA: Positional obstructive sleep apnoea
POSA: Positional obstructive sleep apnoea
PPV: Positive predictive value
PSG: Polysomnography
PT: Positional therapy
SDB: Sleep-disordered breathing
SMRC: Sleep Medicine and Research Center
TIB: Time in bed
TST: Total sleep time
UAE: United Arab Emirates
